# Anticoagulants as panacea in COVID-19 infection

**DOI:** 10.1590/1677-5449.200063

**Published:** 2020-06-12

**Authors:** Marcone Lima Sobreira, Marcos Arêas Marques

**Affiliations:** 1 Universidade Estadual Paulista – UNESP, Botucatu, São Paulo, SP, Brasil.; 2 Universidade do Estado do Rio de Janeiro – UERJ, Unidade Docente Assistencial de Angiologia, Rio de Janeiro, RJ, Brasil.; 3 Universidade Federal do Estado do Rio de Janeiro – UNIRIO, Serviço de Cirurgia Vascular, Rio de Janeiro, RJ, Brasil.

The association between viral infections, such as human immunodeficiency virus (HIV), hepatitis C, and influenza, and venous thromboembolism (VTE) is well-established in the medical literature and the scientific community demonstrated this link again during the Chikungunya and Zika epidemics of 2017.^1^^,^^2^ The ongoing global COVID-19 pandemic that started in Wuhan (China) and is caused by the SARS-CoV-2 coronavirus strain has already infected around 310,000 Brazilians and caused more than 20,000 deaths, according to data from the Ministry of Health.^3^

Although it has a wide clinical spectrum, ranging from an asymptomatic form to a severe acute respiratory syndrome (SARS),^4^ what has attracted the attention of angiologists and vascular surgeons are the symptoms related to inflammation of the vascular system and the hypercoagulability, which cause manifestations such as vasculitis of small vessels and micro and macrovascular thrombosis of arteries and/or veins. Another observation that has attracted attention since the outset is the relationship between elevation of D-dimer (DD) and poor disease prognosis,^5^ demonstrating a clear association between exacerbation of the systemic inflammatory response and the resulting prothrombotic state.^6^

As the number of severe COVID-19 cases progressively increased, it was observed worldwide that there were elevated rates of deep venous thrombosis and pulmonary embolism in this subset of patients, even with pharmacoprophylaxis or full anticoagulation that would theoretically be sufficient for hospitalized clinical patients.^7^ In view of the above context, it is to be expected that there will be a progressive increase in publications relating VTE with infection by COVID-19 in the medical literature, aiming to share the as-yet scant knowledge that has been accumulated on this new infection.

However, despite the growing research networks that have formed to investigate COVID-19, it is noteworthy that the majority of studies present weak evidence, because, in general, what has been published to date are guidelines from specialty societies, expert opinions, in vitro studies, case reports, and some cases series (with small sample sizes). Moreover, hand-in-hand with this explosion of publications, we are confronted with a range of theories and normative statements on prophylaxis and treatment for VTE, serial measurement of DD, and administration of anticoagulants at the most varied posologies to these patients, without adequate scientific evidence, albeit because there has not been sufficient time to produce it.

What can be stated with confidence, so far, is that SARS-CoV-2 infection appears to have an elevated thrombogenic potential, with repercussions for pulmonary microcirculation, and so there may be some benefit, which remains to be proved, from systemic anticoagulation.^8^ It is important to remember that, when dealing with anticoagulants, it is always necessary to consider the risk/benefit balance, weighing the potential efficacy: prevention of thrombosis pulmonary microcirculation and also at the arteriole-capillary level,^9^ against the risk of complications, such as bleeding.

Some reports by Chinese authors suggest that clinical improvement of patients infected by SARS-CoV-2 was associated with parenteral anticoagulation, notably low molecular weight heparin (LMWH); however, it is worth mentioning that the lack of criteria for indicating anticoagulant treatment and consequent indiscriminate use of anticoagulation may not be of benefit to patients,^10^ and it is too early to recommend this as the routine conduct in general. The beneficial effects of heparin in these patients (unfractionated heparin [UFH] or LMWH) appear to be multifaceted. In addition to their known anticoagulant and anti-inflammatory effects, heparins appear to play a protective role in endothelium, by antagonizing histones that cause endothelial injury and, as a result, microcirculation damage, and also appear to have an antiviral activity by competing with the virus for the binding site on the cell surface.^11^

We must be very careful not to fall into the panacea of using anticoagulants unchecked, for prophylaxis and treatment of the obvious hypercoagulability and its clinical manifestations that occur in these patients, especially based on serial DD assays, without a foundation in randomized, double-blind, controlled, multicenter clinical trials that can reliably demonstrate the scientific evidence necessary to guide management of this disease and, primarily, of patients. In the absence of such studies, we can and should rely on the existing guidelines for VTE treatment and prophylaxis in clinical patients, since they are evidence-based and valid.

More recently, a panel of experts published a document in which they discuss rationalization of use of anticoagulants in COVID-19 positive patients. They suggest that hospitalized patients should be categorized for risk of VTE in order to then be given the best prophylaxis for each specific case. With regard to continuing prophylaxis (especially chemical prophylaxis) for patients after discharge, there is no evidence on which to base systematic prescription; so it is suggested that patients are once more categorized at discharge for thrombotic and hemorrhagic risk and that the best treatment be chosen on this basis, in addition to instructing all to remain active when confined at home. There are still controversies and arguments with regard to use of intermediate or therapeutic heparin doses in these patients: the majority recommend use of prophylactic doses, whereas a minority consider that it is reasonable to use full or intermediate doses in this subset of patients.^12^

Knowledge with respect to this disease’s response to any type of suggested treatment is extremely fluid and concepts are constantly being renewed constantly, so great judgment and care are needed to manage it, attempting to always keep in mind a palpable and solid scientific basis, to avoid harming the patient.
