# Supplementary material for: Anticoagulants as panacea in COVID-19 infection
Source: J Vasc Bras. 2020 Jun 12;19:e20200063. doi: 10.1590/1677-5449.200063 (PMC8202164; doi:10.1590/1677-5449.200063)
Supplement: Translation

# Translation: A panaceia dos anticoagulantes na infecção pela COVID-19

**Authors:** Marcone Lima Sobreira, Marcos Arêas Marques

**Affiliations:** 1 Universidade Estadual Paulista – UNESP, Botucatu, São Paulo, SP, Brasil.; 2 Universidade do Estado do Rio de Janeiro – UERJ, Unidade Docente Assistencial de Angiologia, Rio de Janeiro, RJ, Brasil.; 3 Universidade Federal do Estado do Rio de Janeiro – UNIRIO, Serviço de Cirurgia Vascular, Rio de Janeiro, RJ, Brasil.

A associação entre infecções virais, como a do vírus da imunodeficiência humana (HIV), hepatite C e influenza, e o tromboembolismo venoso (TEV) já está bem estabelecida na literatura médica e já havia voltado a ser evidência na comunidade científica na epidemia de Chicungunha e Zika ocorrida em 2017^1^^,^^2^. A atual pandemia mundial de COVID-19, iniciada em Wuhan (China), causada pela cepa SARS-CoV-2 do coronavírus já infectou cerca de 310.000 brasileiros, com mais de 20.000 mortes aproximadamente, segundo dados do Ministério da Saúde^3^.

Apesar de seu amplo espectro clínico, que varia desde a forma assintomática até uma síndrome respiratória aguda grave (SARS)^4^, tem chamado atenção dos angiologistas e dos cirurgiões vasculares os sintomas relacionados à inflamação do sistema vascular e à hipercoagulabilidade que levam a manifestações como vasculite de pequenos vasos e trombose micro e macrovascular de artérias e/ou veias. Outro dado que chamou atenção desde o início foi a relação entre a elevação do dímero-D (DD) e o mau prognóstico da doença^5^, demonstrando uma clara associação entre o agravamento do quadro inflamatório sistêmico e o estado pró-trombótico resultante^6^.

Com o aumento progressivo dos números de casos graves da COVID-19, houve uma constatação mundial da alta incidência de trombose venosa profunda e embolia pulmonar nesse perfil de pacientes, mesmo com a farmacoprofilaxia ou anticoagulação plena teoricamente adequada para pacientes clínicos internados^7^. Nesse contexto descrito anteriormente, é de se esperar que haja um aumento progressivo de publicações relacionando o TEV à infecção por COVID-19 na literatura médica, visando compartilhar o ainda pequeno conhecimento sobre essa nova infecção.

Entretanto, apesar da crescente rede de pesquisas que se criou em torno da COVID-19, nota-se que a maioria desses estudos tem evidência fraca, pois o que se tem até o momento são, de uma forma geral, diretrizes de sociedades de especialidades, opiniões de especialistas, estudos *in vitro*, relatos de casos e algumas séries de casos (com tamanho amostral reduzido). Além disso, junto a essa explosão de publicações, nos deparamos com diversas teorias e normatizações a respeito da profilaxia e tratamento do TEV, mensurações seriadas do DD e o uso de anticoagulantes nas mais variadas posologias nesses pacientes, sem evidências científicas adequadas, até pela falta de tempo hábil para produzi-las.

O que se pode elucubrar de fato, até o momento, é que a infecção pelo SARS-CoV-2 parece carregar potencial trombogênico aumentado, com repercussões em microcirculação pulmonar, podendo haver algum benefício, ainda a ser comprovado, de anticoagulação sistêmica^8^. É importante ressaltar que, em se tratando de anticoagulantes, é necessário sempre contrabalançar o binômio risco/benefício, pesando a sua potencial eficácia: prevenção de trombose em microcirculação pulmonar e, também, em território arteriolocapilar^9^ contra o risco de complicações, como o sangramento.

Alguns relatos de autores chineses sugerem a melhora clínica de pacientes infectados pelo SARS-CoV-2 com o uso parenteral de anticoagulação, notadamente a heparina de baixo peso molecular (HBPM); entretanto, vale a ressalva que a falta de critério na indicação de terapia anticoagulante, com consequente uso indiscriminado de anticoagulação, pode não trazer benefício aos pacientes^10^, sendo muito temerário estabelecer como protocolo de conduta de uma forma generalizada. O efeito benéfico da heparina nesses pacientes [HNF (heparina não fracionada) ou HBPM] parece ser multifacetado. Além dos conhecidos efeitos anticoagulante e anti-inflamatório, as heparinas parecem ter papel como protetor endotelial, por antagonizar as histonas que causam injuria endotelial e, portanto, microcirculatória, e um efeito antiviral por competir com o vírus pelo sítio de ligação da superfície celular^11^.

Temos que ter muito cuidado para não cair na panaceia do uso de anticoagulantes de forma desenfreada, na profilaxia e no tratamento da evidente hipercoagulabilidade e suas manifestações clínicas que ocorrem nesses pacientes, especialmente apoiado em dosagens seriadas do DD, sem nos basearmos em ensaios clínicos multicêntricos, randomizados, duplos-cegos e controlados que possam atestar com confiabilidade a evidência científica necessária ao balizamento de condutas frente à doença e, principalmente, frente ao doente. Na falta desses estudos, podemos e devemos nos apoiar nas diretrizes existentes para tratamento e profilaxia do TEV em pacientes clínicos, pois elas são embasadas e validadas.

Mais recentemente, um painel de especialistas publicou um documento em que se discute, também, a racionalização do uso de anticoagulantes em pacientes COVID-19 positivos. Os autores sugerem que o paciente internado deva ser categorizado quanto ao risco de TEV para, então, receber a melhor profilaxia para cada caso específico. Em relação à extensão da profilaxia (especialmente a química), para pacientes no pós-alta, ainda não há subsídios que suportem a prescrição sistemática; sugere-se que os pacientes também sejam categorizados na alta quanto ao risco trombótico e hemorrágico, sendo, dessa forma, direcionados à melhor terapêutica, devendo ser todos orientados a se manter ativos enquanto confinados em ambiente doméstico. No que concerne à utilização de doses intermediárias ou terapêuticas de heparina para esses pacientes, ainda há controvérsia e polêmica: a maioria considera utilizar dose profilática, enquanto uma minoria acha razoável a utilização de dose plena ou intermediária para esse nicho de pacientes^12^.

O conhecimento a respeito da resposta dessa doença a qualquer tipo de tratamento sugerido está extremamente volúvel com renovação de conceitos diuturnamente, sendo necessários muito critério e parcimônia na tomada de conduta, procurando sempre ter em mente base científica palpável e sólida para não acarretar danos ao paciente.
